# Periostin and Discoidin Domain Receptor 1: New Biomarkers or Targets for Therapy of Renal Disease

**DOI:** 10.3389/fmed.2017.00052

**Published:** 2017-05-09

**Authors:** Niki Prakoura, Christos Chatziantoniou

**Affiliations:** ^1^Institut National de la Santé Et de la Recherche Médicale UMRS 1155, Tenon Hospital, Paris, France; ^2^Sorbonne Universités, UPMC Paris 6, Paris, France

**Keywords:** chronic kidney disease, biomarkers, therapeutic targets, periostin, discoidin domain receptor 1

## Abstract

Chronic kidney disease (CKD) can be a life-threatening condition, which eventually requires renal replacement therapy through dialysis or transplantation. A lot of effort and resources have been invested the last years in the identification of novel markers of progression and targets for therapy, in order to achieve a more efficient prognosis, diagnosis, and treatment of renal diseases. Using experimental models of renal disease, we identified and studied two promising candidates: periostin, a matricellular protein with high expression in bone and dental tissues, and discoidin domain receptor 1 (DDR1), a transmembrane collagen receptor of the tyrosine kinase family. Both proteins are inactive in physiological conditions, while they are highly upregulated during development of renal disease and are primarily expressed at the sites of injury. Further studies demonstrated that both periostin and DDR1 are involved in the regulation of inflammation and fibrosis, two major processes implicated in the development of renal disease. Targeting of either protein by genetic deletion or pharmacogenetic inhibition via antisense oligonucleotides highly attenuates renal damage and preserves renal structure and function in several animal models. The scope of this review is to summarize the existing evidence supporting the role of periostin and DDR1 as novel biomarkers and therapeutic targets in CKD.

## Introduction

Chronic kidney disease (CKD) represents a major burden for modern societies affecting almost 10% of the population worldwide, while its growing incidence accounts for the doubling of the global number of deaths caused by CKD over the past 25 years (1). Diabetes, hypertension, and primary glomerular diseases are major causes of CKD. Despite their different origin, these diseases are characterized by common mechanisms like chronic inflammation and development of fibrosis, which lead to the impairment of tubulointerstitial, glomerular, and vascular compartments of the kidney causing gradual loss of renal function.

During the last decades, the development of systems biology approaches in combination with mouse genetics tools led to significant progress in our understanding of the complex mechanisms orchestrating these processes, with the identification of a plethora of novel mediators and pathways participating in the progression of CKD. However, no efficient treatment has been shown yet to arrest or reverse the course of human CKD, which upon progression to end-stage renal disease will eventually require renal replacement therapy. Therefore, identification of novel biomarkers and/or targets for therapy is critical for the earlier diagnosis or treatment of CKD patients and represents a major research topic in the field of renal diseases (2–5).

Among the oldest and most acknowledged mediators of renal disease are angiotensin II (Ang-II), transforming growth factor-β (TGF-β), platelet-derived growth factors (PDGFs), connective tissue growth factor (CTGF), endothelin-1 (ET-1), and key pro-inflammatory cytokines (e.g., MCP-1, TNF-α). Inhibition of these factors in animal models was shown to efficiently ameliorate the progression of renal disease by limiting renal inflammation and fibrosis. However, only renin-angiotensin system (RAS) antagonists are presently applied in clinical practice with limited potential in cases of human CKD, while drugs targeting some of the above mediators have come up to clinical trials but there is no certainty that they will be finally approved for use in patients (6, 7).

Our team has recently identified two novel mediators of renal disease which may serve as promising biomarkers and/or targets for therapy of CKD: periostin and discoidin domain receptor 1 (DDR1). This review will summarize the latest literature on these proteins focusing on renal diseases and discuss their potential of constituting the basis of future treatment against CKD.

## Periostin

Periostin is a 90-kDa secreted matricellular protein originally identified because of its high expression in periosteum and periodontal ligament (8). Although periostin is highly expressed during development, its expression is compromised in mature tissues, while it is considerably induced in tissue injury and remodeling conditions.

The protein is composed of three distinct regions serving different functions: an N-terminal cysteine-rich EMI domain reported to bind to collagen I, fibronectin and Notch 1, a tandem repeat of four fasciclin-I domains containing binding sites for BMP-1, tenascin-C, as well as several integrins, and a C-terminal domain which is a site of proteolytic cleavage and splice variant formation (9–13). The ability of periostin to interact with extracellular matrix (ECM) components and cell-surface receptors renders the protein capable to modulate both the biomechanical properties of connective tissues and the cell-matrix interactions, thus regulating important processes like cell adhesion, migration, proliferation, and differentiation.

Periostin has been shown to play important roles in the pathophysiology of several organs. In the heart, periostin production by fibroblasts was demonstrated to be crucial for collagen fibrilogenesis and cardiac healing short-term after myocardial infarction, whereas in chronic cardiac disease models periostin null mice were protected from hypertrophy and fibrosis (14, 15). Studies in animal models and patients with idiopathic pulmonary fibrosis or asthma indicated periostin as an important mediator and prognostic factor of lung diseases (16–18). Of note, serum periostin levels of asthmatic patients were proven to reflect the response to therapy in two independent clinical studies (19, 20). Moreover, periostin was shown to be a critical player in cancer progression and metastasis, by promoting the recruitment of tumor-associated macrophages in the cancer tissue or by interacting with and permitting the colonization of cancer cells (21, 22). Elevated periostin expression was also detected in biopsies of renal cell carcinoma (RCC) and was associated with increased tumor aggressiveness and poor prognostic survival (23). In another study, periostin was found to be produced by the stroma cells of both organ-confined and metastasized RCCs, enhancing epithelial tumor cell attachment (24).

## Periostin in CKD

Over the last years, accumulating evidence has highlighted the involvement of periostin in animal and human CKD. A first study aiming to identify differentially expressed transcripts in patients with glomerular diseases unraveled periostin as the most highly expressed target among matricellular proteins (25). Accordingly, using the model of L-NAME-induced hypertensive nephropathy, we identified periostin as one of the most highly expressed genes in a transcriptomic analysis. Interestingly, periostin levels were closely correlated with the disease progression or regression upon treatment with angiotensin type I receptor antagonists, as well as with classical indexes of renal function like creatininemia, proteinuria, and renal blood flow (26). These results were recently confirmed in another model of hypertensive nephrosclerosis (27). In parallel, periostin was found *de novo* expressed in biopsies from patients with various renal diseases, including diabetes, lupus nephritis, IgA nephropathy, and focal segmental glomerulosclerosis. The protein was over-expressed in areas with periglomerular or interstitial fibrosis and its expression levels were associated with the degree of histological damage and the decline of glomerular filtration rate (25, 28–31). Several studies also reported the detection of elevated periostin levels in the urine of CKD patients, which were correlated with the stage of the disease and could predict worsening renal outcomes (29–32).

Subsequent studies by our group investigated the role of periostin in renal disease. Mice with genetic deletion of periostin showed substantially reduced inflammation and fibrosis in the models of unilateral ureteral obstruction (UUO) and nephrotoxic serum (NTS)-induced glomerulonephritis (33, 34). Most importantly, by using administration of antisense oligonucleotides against periostin in a pharmacogenetic approach, we showed that inhibition of periostin after the establishment of proteinuria could ameliorate the progression of the disease and preserve renal structure and function (34). In another study, periostin was found over-expressed in renal cyst-lining epithelial cells from patients with polycystic kidney diseases (PKD), while periostin null mice were protected in a PKD mouse model, showing reduced cyst number and size, less interstitial fibrosis, and improved renal function (35).

Several fibrotic or inflammatory mediators were shown to induce the expression of periostin *in vitro* or *in vivo* in different disease contexts. The pro-fibrotic growth factor TGF-β1 is a known potent inducer of periostin expression (8, 36, 37). Ang-II was shown to upregulate periostin in cardiac fibroblasts or vascular smooth muscle cells through a complex network involving Ras/CREB and ERK/TGF-β1 or PI3 kinase pathways, respectively (37, 38). More recently, PDGF-B was demonstrated to induce periostin expression in renal mesangial cells, associated with cell proliferation and matrix production (39). The interleukins, IL-4 and IL-13, have been associated with induction of periostin in bronchial asthma (16, 40). Moreover, we have recently demonstrated that periostin is induced by NFκB and other pro-inflammatory transcription factors in experimental glomerulonephritis (34).

**Figure 1:**
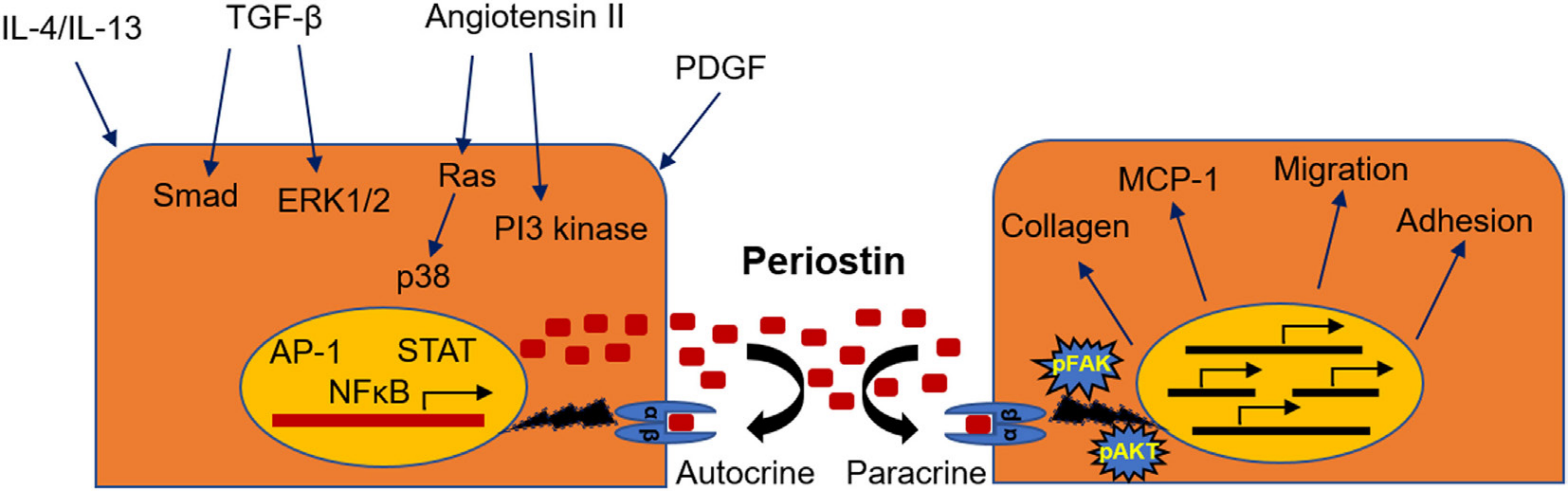
**Mechanisms of induction and physiopathological actions of periostin during renal disease**. Periostin can be induced by a variety of different growth factors, transcription factors, or signaling pathways (left), while its activation leads to stimulation of integrin signaling, matrix assembly, promotion of inflammatory pathways, and cell phenotype changes (right).

The mechanisms through which periostin regulates disease development have been described to some extent, although they may differ from one setting to another and there is still incomplete understanding to be further elucidated. The interaction of periostin with collagen and other ECM components assists to the cross-linking and incorporation of collagen into the ECM, which promotes the expansion of fibrosis (9, 11, 12). On the other hand, periostin transmits signals inside the cells through interactions with cell-surface integrin receptors such as αvβ3 and αvβ5. This interaction results in activation of the PI3 kinase/Akt and focal adhesion kinase pathways, promoting cell adhesion, migration, and differentiation. In this context, periostin was shown to promote adhesion and migration of cancer cells (13), vascular smooth muscle cells (41), and mesenchymal stem cells (42) or facilitate the infiltration of macrophages into the cancer tissue (21). Moreover, periostin may play a critical role as mediator of the inflammatory process. In chronic allergic skin inflammation, periostin was shown to promote Th-2 type immune responses (43). In another study, lung fibroblasts isolated from periostin null mice had impaired production of chemokines and inflammatory cytokines in response to TNF-α (17). Besides, we also demonstrated that mice lacking periostin exhibit highly attenuated immune responses during development of renal disease (33, 34). The mechanisms of activation and the possible role of periostin in CKD are depicted in Figure 1.

## Discoidin Domain Receptor 1

Discoidin domain receptor 1 is a transmembrane tyrosine kinase receptor of both fibrillar and non-fibrillar collagens, with a wide body distribution and a predominant expression in epithelial cells. The protein is composed of three different regions with distinct functions: an extracellular discoidin homology domain that comprises the collagen-binding site, a transmembrane domain that mediates the receptor dimerization, and a large intracellular region that contains several tyrosine residues that can be phosphorylated and possesses a tyrosine kinase activity. The mechanism of DDR1 activation involves collagen binding to pre-formed receptor dimers followed by slow tyrosine autophosphorylation and receptor activation which may, however, persist for several hours or days post stimulation (44, 45).

Discoidin domain receptor 1 has been shown to regulate a wide variety of cell functions, including migration, survival, proliferation, and ECM remodeling, in a cell type- and context-dependent manner. DDR1 was reported to promote the mitogen-activated protein kinase (MAPK) signaling through activation of either the ERK1/2 or JNK pathways in different cell lines. It can also induce the expression of matrix metalloproteinases in vascular smooth muscle, bronchial epithelial, and cancer cells. Moreover, DDR1 activation was shown to induce the PI3 kinase/Akt and NFκB pathways in cancer cells (45). In addition to mediating direct collagen-stimulated signaling, DDR1 can interact with other receptors modulating their functions. For example, DDR1 was shown to either promote or counteract integrin α2β1-mediated signaling in different contexts and to bind and activate Notch1 promoting survival of cancer cells (44, 46).

Deregulated function of DDR1 has been described in various human diseases, including several types of cancer, atherosclerosis, osteoarthritis, and fibrotic diseases. DDR1 was shown to be pro- or anti-tumorigenic in different types of cancer. More specifically in renal cancer, phospho-DDR1 (pY792/6) was found to be preferentially enriched in papillary RCC compared to clear cell RCC samples (47). On the other hand, in another study, the expression level of DDR1 was correlated with the tumor stage and events of lymph node metastasis in clear cell RCC patients and was associated with increased migration and invasion of RCC cell lines (48). In animal models of atherosclerosis, DDR1 null mice showed a reduction in atherosclerotic lesions, which was associated with a pro-inflammatory effect of DDR1 by promoting macrophage infiltration (44, 45). Moreover, DDR1-deficient mice exhibited reduced pulmonary inflammation and fibrosis induced by bleomycin administration, accompanied by decreased activation of p38 MAPK (49).

## DDR1 in CKD

**Figure 2:**
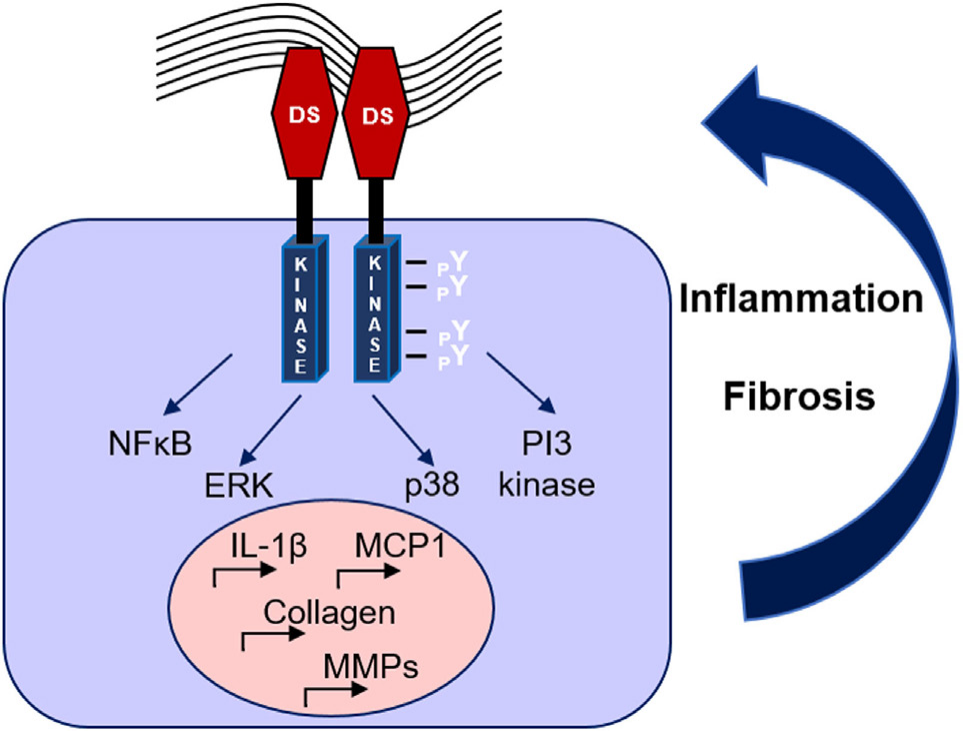
**Mechanisms of discoidin domain receptor 1 (DDR1) activation and amplification of renal damage during chronic kidney disease**. Collagen binding to DDR1 receptor dimers induces the receptor phosphorylation and activation, which stimulates pro-inflammatory and pro-fibrotic pathways creating a vicious circle of continuous renal damage.

Recent studies have demonstrated DDR1 as an important mediator in renal diseases. A first study by our team in the model of Ang-II-induced hypertensive nephropathy revealed that DDR1-deficient mice were markedly protected against the development of proteinuria, glomerular and perivascular fibrosis, and inflammation (50). Subsequent data obtained from studies in the UUO model indicated that DDR1 is an effector of the inflammatory response in renal disease, since macrophages isolated from DDR1 null mice showed impaired migration toward MCP-1 (51). In accordance with these results, elevated DDR1 expression was detected in stimulated peripheral blood mononuclear cells and activated leukocytes (45). In a mouse model of severe glomerulonephritis, both deletion and antisense treatment against DDR1 attenuated the renal damage. In this study, DDR1 was *de novo* expressed in damaged podocytes, while it was also found increased in the glomeruli of patients with lupus nephritis and good pasture syndrome (52). Most interestingly, inhibition of DDR1 with antisense oligonucleotides after establishment of the disease was protective in models of glomerulonephritis and ureteral obstruction (53). In a mouse model of Alport syndrome, a genetic disorder caused by an inherited defect in type IV collagen, genetic deletion of DDR1 attenuated the development of renal fibrosis by reducing the renal content of pro-inflammatory and pro-fibrotic cytokines and by decreasing ECM deposition (54). Interestingly, a recent study in the remnant kidney model demonstrated that both the collagen-binding site and the kinase domain of DDR1 are necessary for the receptor-mediated collagen production, since mutation in either site decreased DDR1-induced collagen deposition by mesangial cells (55). Furthermore, single nucleotide polymorphisms (SNPs) of yet unknown function in coding sites and the 3′-untranslated region of the DDR1 gene were associated with increased susceptibility and pathological advancement of childhood IgA nephropathy in a large population study (56). The described mechanisms through which DDR1 may regulate renal dysfunction are illustrated in Figure 2.

## Potential of Periostin and DDR1 as Biomarkers or Therapeutic Targets in CKD

Over the last years, a lot of effort has been made in identifying novel markers and therapeutic targets for CKD, an incurable to date pathology. The new molecules promise an earlier diagnosis or a more efficient treatment of the disease, which would facilitate the life of CKD patients and unburden the public health care system. However, several promising candidates, although valuable in animal models were proven ineffective in human pathology, while others despite playing significant roles during the course of the disease, do not meet the criteria for constituting efficient novel biomarkers or targets for therapy.

An ideal biomarker should be expressed early during the progression of renal disease, should be easily detectable in biological fluids like plasma or urine, and should correlate well with the different stages of CKD development. Periostin fulfils several of these criteria according to the existing data. Its expression is low in healthy kidneys, while it is highly induced in several models of renal disease (UUO, hypertensive nephropathy, NTS-induced glomerulonephritis, PKD) and its expression levels correlate very well with the stage of the pathology and the decline of renal function (26, 33–35). Moreover, periostin is secreted and can be easily detected in a non-invasive manner in plasma or urine samples. The normal levels of periostin in the plasma of healthy donors range between 10 and 60 ng/ml (57, 58), while in urine it is less than 1 ng/mg creatinine (29, 31, 32). Of note, although its expression in normal urine is negligible, it was shown to substantially increase and correlate with the severity of the disease in several types of CKD (27–30). It remains to be elucidated whether periostin may represent an earlier and more specific marker for renal disease or a subgroup of CKD patients compared to the existing standards. DDR1, as an integral membrane receptor, cannot be an easily detectable biomarker in biological fluids as periostin. Presently, DDR1 has been exclusively used as a tissue biomarker in the disease setting. However, cleavage of the extracellular domain of DDR1 by cell membrane proteases, a regulatory mechanism known as “ectodomain shedding,” has been largely described in cell culture systems (45). If this is also confirmed *in vivo* in the disease context, the cleaved ectodomain of DDR1 could be detectable and usable as a plasma or urine biomarker in renal diseases.

In terms of therapy, a promising target should be easily accessible by potential drugs, while its silencing or inhibition in animal models of CKD should preserve renal structure and function. In this context, both periostin and DDR1 may represent potential targets for therapy of renal disease. The expression of periostin is primarily localized at the sites of injury both in animal models and human biopsies of CKD, which demonstrates that periostin is highly associated with renal damage. For example, periostin was found to be produced by vascular cells in hypertensive nephropathy, by tubular epithelial cells in ureteral obstruction and PKD, and by glomerular podocytes and parietal epithelial cells in glomerulonephritis (25, 26, 33–35). Moreover, mice lacking periostin or treated with antisense oligonucleotides against periostin are protected from the progression of severe forms of CKD [(26, 33–35); Table 1]. There are three potential strategies for periostin targeting in renal disease: (1) blocking antibodies as neutralizing agents of periostin function, (2) inhibition of periostin expression with antisense nucleotides, and (3) specific inhibitors of the interaction between periostin and integrins to block the downstream signaling. The first two approaches have been used by several researchers including us in animal models of different diseases to show that periostin has the potential of being a target for therapy (18, 22, 33, 34). The construction of specific blockers of the interaction between periostin and its receptors has not been described yet and may require an in-depth knowledge of the exact interaction sites and residues. Currently, several investigators are developing the necessary tools for application in humans (specific blocking peptides/antibodies, antisense with increased stability) which will verify the therapeutic potential of periostin targeting in human CKD.

**Table 1:** **Functional roles of periostin and discoidin domain receptor 1 (DDR1) in experimental chronic kidney disease**.

Protein	Animal model	Functional role	Reference
Periostin	L-NAME-induced hypertensive nephropathy	Correlation with parameters of renal function, treatment with periostin antisense protects against vascular hypertrophy, glomerulosclerosis, perivascular fibrosis, and tubular dilation	(26, 33)
	Unilateral ureteral obstruction (UUO)	Knock-out mice are protected against renal inflammation and fibrosis	(33)
	NTS-induced glomerulonephritis	Periostin induced by NFκB activates the integrin avβ3 signaling pathway to mediate inflammation and fibrosis accompanied by deterioration of renal structure and function	(34)
	*Pcy/pcy* polycystic kidney disease	Periostin promotes cyst epithelial cell proliferation and interstitial fibrosis mediated by activation of the mTOR pathway	(35)
	MRL/*lpr* lupus nephritis	Periostin mediates mesangial cell proliferation and extracellular matrix production downstream of platelet-derived growth factor and PI3 kinase	(39)
DDR1	Angiotensin II-induced hypertensive nephropathy	Knock-out mice are protected against periglomerular and interstitial fibrosis, inflammation, and proteinuria	(50)
	UUO	DDR1 promotes macrophage migration enhancing renal inflammatory cell infiltration and fibrosis	(51)
	NTS-induced glomerulonephritis	Genetic or pharmacogenetic inhibition of DDR1 preserves renal structure and function through reduction of the inflammatory response	(52)
	NTS-induced GN, UUO	Inhibition of DDR1 expression with antisense after initiation of renal disease delays or arrests the progression of the pathology	(53)
	Alport syndrome (Col4a3 KO)	DDR1 promotes renal inflammation and fibrosis through signaling via transforming growth factor-β, connective tissue growth factor, NFκB, and IL-6	(54)

Discoidin domain receptor 1 is also over-expressed and activated in several animal or human renal pathologies, while its inhibition even after the establishment of renal damage attenuates renal inflammation and fibrosis and preserves kidney function [(50–54) and Table 1]. Appealing approaches for DDR1 targeting would be either to block the interaction of DDR1 with collagen or inhibit the tyrosine kinase activity of DDR1 in order to prevent downstream signaling. Small-molecule tyrosine kinase inhibitors are classified in type I which target the catalytically active conformation of the kinase, and type II that target the inactive conformation of the receptor which allows additional interactions with the receptor and increases the selectivity of the inhibitor. Known tyrosine kinase inhibitors (dasatinib, imatinib, nilotinib) initially identified as blockers of the tyrosine kinase BCR-ABL for potential use in cancer, display also the ability to block collagen-mediated DDR1 autophosphorylation. However, the lack of specificity of these inhibitors for DDR1 increases the possibility for off-target effects and limits their clinical application against DDR1. Interestingly, tyrosine kinase inhibitors selective for DDR1 were identified and proven efficient in inhibiting the receptor autophosphorylation and the proliferation of cancer cells highly expressing DDR1 (44). Moreover, the continual screening of existing libraries of chemical compounds may lead to the identification of novel-specific and more effective DDR1 inhibitors. Eventually, these inhibitors could be easily tested in animal models of CKD for their efficacy to inhibit renal disease.

## Conclusion and Perspectives

We identified periostin and DDR1 as novel promising targets for therapy which could be applied in the future for the prevention and/or treatment of patients with CKD. Our observations in biopsies and animal models of CKD demonstrated that both periostin and DDR1 are expressed in low levels under physiological conditions, are highly activated after renal damage and expressed primarily by injured cells, are well correlated with CKD progression, while their deletion or pharmacogenetic inhibition largely protects from development of renal disease. Despite the recent advances, several aspects of the mechanisms of action of periostin and DDR1 are still elusive. The most crucial limitation for pursuing the targeting of these new candidates in human CKD is the current lack of validated tools available for use in humans (monoclonal antibodies, specific blockers, elisa kits); however, we are confident that this limitation will be surpassed in the near future as the field is rapidly expanding, which will hopefully allow a most efficient prognosis and treatment of CKD.

## Author Contributions

NP reviewed the literature and prepared the manuscript. CC supervised and reviewed the manuscript.

## Conflict of Interest Statement

The authors declare that the research was conducted in the absence of any commercial or financial relationships that could be construed as a potential conflict of interest.
